# Depression and Its Association with Parental Neglect among Adolescents at Governmental High Schools of Aksum Town, Tigray, Ethiopia, 2019: A Cross Sectional Study

**DOI:** 10.1155/2020/6841390

**Published:** 2020-04-30

**Authors:** Enguday Tirfeneh, Mengesha Srahbzu

**Affiliations:** ^1^Aksum University, College of Health Science, Department of Psychiatry, Aksum, Ethiopia; ^2^University of Gondar, College of Medicine and Health Science, Department of Psychiatry, Gondar, Ethiopia

## Abstract

**Background:**

Depression is one of the most serious and prevalent mental illnesses that can result in serious disability and ending life by committing suicide and homicide. The risks of having depression are substantially higher in persons who have parental neglect when compared to the general population.

**Objective:**

To detect prevalence of depression and its association with parental neglect among adolescents in governmental high schools at Aksum town, Tigray, Ethiopia, 2019.

**Method:**

A facility-based cross-sectional study was conducted at Aksum town high schools. A simple random sampling technique was applied. Data was collected with face-to-face interview. Data was analyzed using IBM Statistical Package for Social Science version 22. Bivariate and multivariate logistic regressions were done. Adjusted odds ratio at a *p* value < 0.05 with 95% confidence interval was taken to declare statistical significance of variables.

**Result:**

A total of 624 students were asked to participate with a response rate of 99.05%. Prevalence of depression was found to be 36.2%. Depression among adolescents was found to have significant and strong association with parental neglect (AOR = 2.61, 95% CI 1.83, 3.72). *Conclusion and Recommendation*. In the current study, the prevalence of depression is found to be high when compared to other populations. Significant and strong association is also determined between parental neglect and depression. It is good if teachers give emphasis for those students who seem psychologically unwell. It is good if Aksum University comprehensive hospital starts a campaign which will teach about the effect of parental neglect on the adolescents' mental health.

## 1. Introduction

Adolescence (10–19 years) is among the age group which highly experiences several factors in their day-to-day activities [1]. Adolescent age group is also a time that individuals will experience physical changes and take different responsibilities which may give rise to get mental disorders as it is a new environment for them. Youths constitute almost one-third of the world's population, and among them, one-third live in the developing world, where they form up to half of the population [2].

Child or adolescent abuse is an issue concerning millions of youths and their families worldwide. Maltreatment of youths can be defined as an act of omission or commission by others who are caregivers that may have danger, possibility of danger, or threats of danger to adolescents [3]. Parental neglect involves an act of omission, and it is defined as a failure by a caregiver to address the adolescents' basic physical, emotional, medical/dental, or educational needs [4, 5].

The burden of psychiatric disorders on youths is enormous and covers a great number of people in all types of societies. The majority of psychiatric disorders begin at an early age (12–24), even though they are supposed to be experienced in older individuals later in life. Depression is the most common and severe psychiatric disorder that leads to magnifying problems in an adolescent's capacity to take care of his or her everyday responsibilities and functionalities. Depressive disorders in adolescents result in a horrible consequences even to other healthy components. It may lead to educational impairment, comorbid psychoactive substance abuse, behavioral difficulties, and risky reproductive and sexual practices [6].

A mental health service for adolescents' mental health problems is not adequate yet, even in the developed world. Age-related stigma is among the major contributory factors for unmet mental health needs in youths [7]. Currently, depression is recognized as the first psychiatric disorder of youths which is related to its common presentation, episodic nature, and its ability to cause significant complications and impairment. According to the 2009 discussion paper released by the World Health Organization (WHO), among 66 million individuals experiencing depression, 85% live in the developing world [8].

Some statistics indicate that depression is as frequent as 20% common in adolescents [9] and 43.4% of high school students in Tehran to be depressed [10]. A study which was conducted between China and America using the Chinese version of the Beck Depression Inventory reported that 15% of participants had depression among 503 subjects [11]. Another study in New York revealed the magnitude of depression in high school students to be 34% [12]. Another survey, using a summarized self-administered Beck's questionnaire, reported severe depression in 18% of 8206 adolescents [13].

A school-based cross-sectional survey was conducted in South India to estimate the prevalence of depression among school-going adolescents. In this, a total of 2432 school-going adolescents were included in the study and 25% of them have been found to have depression [14].

Though depression is one of the major diseases that cause failure to socialize among youths, it is often neglected and has not been given adequate attention it needs. The scarcity services to emotional and other mental problems of children and adolescents make this study necessary for strong emphasis to be given for the support of mental health in Ethiopia for the sake of mental and behavioral welfare of growing children and adolescents [15].

## 2. Materials and Methods

### 2.1. Study Design, Period, and Setting

We conducted a facility-based cross-sectional study from January 1 to 30/2019. The study targeted adolescents at Aksum town high schools. Aksum is located in the Tigray region which is 1024 km far from Addis Ababa. There are three high schools in Aksum town currently named Aksum secondary school, Atse/Kaleb secondary school, and Kedamay Minilik secondary school, and there are a total of 2579 grade nine and 2241 grade ten students in the three high schools.

### 2.2. Sample Size Calculation and Sampling Procedure

We calculated the sample size using a single population proportion formula, and we took the following assumptions: 95% confidence interval and 4% marginal error. The proportion of depression is 39.3% from the previous study [16] with a nonresponse rate of 10%. The final sample size was taken to be 630. All governmental high schools found at Aksum town were included in our study. Students were accessed from each high school proportionally to its total number of students. Sampling frames were prepared in alphabetical basis for each level of grades. Then, a random sampling technique (i.e., lottery method) was applied to select proportionally allocated number of participants from each level of grades. Finally, 285 females and 339 males were selected.

### 2.3. Data Collection Instrument and Techniques

Data was collected by face-to-face interview. Initially, screening tools and other developed structured questionnaire in English language were translated to Amharic and Tigrigna and back to English by independent person to check for consistency and understandability of the tool. Data was collected by six bachelor degree holder health professionals. The data collection process was supervised by the principal investigators. Training was given for data collectors for two days by the principal investigator regarding the process and techniques of data collection.

PHQ-9 was used to assess depression which is a multipurpose instrument for screening, diagnosing, monitoring, and measuring the severity of depression. PHQ 9 score of greater than or equal to 10 has sensitivity and a specificity of 88% and Cronbach's alpha reliability of 0.77 for major depression. Adverse Childhood Experience Questionnaire (ACEQ) which is a 10-item screening tool was used to access parental neglect. ACEQ includes questions that assess emotional abuse and neglect, physical abuse and neglect, educational and medical neglect, and sexual abuse [17]. ACEQ was checked for reliability test, and it has been found to have Cronbach's alpha of 0.83 to assess parental neglect for this study.

The Oslo 3 Social Support Scale was applied to know the level of social support towards adolescents. The scale divides the level of social support into three as poor social support (3-8), moderate social support (3-14), and strong social support (12-14) (reliability of Cronbach's *α* = 0.91) [18]. For this study, its Cronbach's alpha has been found to be 0.84.

### 2.4. Operational Definitions

*Adolescent*: for this study, this means a school-attending person specifically within 15-19 years of age.

*Depression*: this refers to those whose score is greater than 5 from the PHQ-9 scale [19].

*Parental neglect*: this refers to ACEQ which is a self-report instrument covering 10 items, to rate the severity of emotional abuse and neglect, physical abuse and neglect, and sexual abuse [17].

*Social support*: according to the Oslo-3 Social Support Scale, a score of 3-8 is taken as poor support, 9-11 as moderate support, and 12 and 14 as strong support [18].

### 2.5. Data Analysis and Interpretation

After the questionnaire was checked for cleanliness, data was entered using EpiData 3.1 and exported to IBM SPSS version 22 statistical software for analysis. Sociodemographic characteristics of respondents were analyzed by descriptive statistics and presented in percentage, mean, and standard deviations. Bivariate analysis was used to see the association between outcome and independent variables. Multivariate logistic regression was done for those variables whose *p* value is less than 0.2. The significance of variables was considered at a *p* value < 0.05 and 95% CI of their respective adjusted odds ratios.

## 3. Results and Discussion

### 3.1. Sociodemographic Characteristics

A total of 624 participants with a response rate of 99.05% were included in the study. Among this, 339 (54.3%) were females. The age of the majority of students (246 (39.4%)) were known to be 15 years followed by 16 years of age (217 (34.8%)), and more than half (328 (52.6%)) were grade 9 students. More than three-fourths of participants (494 (79.2%)) were Orthodox Christian religion followers (see Table 1).

### 3.2. Social Support-Related Variables

**Figure 1:**
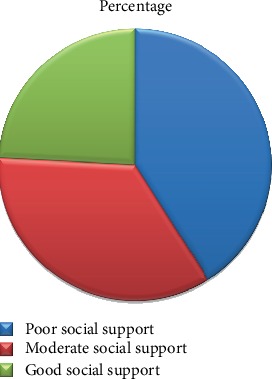
Distribution of the level of social support among high school students at Aksum town, 2019 (*n* = 624).

Among participants, the level of social support was measured. Based on the result of this study, majority of students (256 (41%)) have been found to have poor social support followed by moderate social support (217 (34.8%)) and only 151 (24.2%) students were under good social support (see Figure 1).

### 3.3. Substance-Related Variables

**Figure 2:**
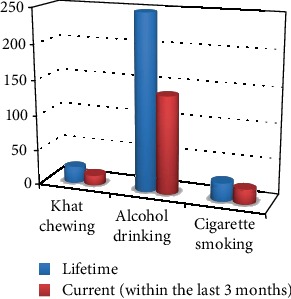
Distribution of substance-related factors among high school students at Aksum town, 2019 (*n* = 624).

Regarding substance use among high school students at Aksum town, only 22 (3.5%) have chewed khat within their lifetime whereas only 14 (2.2%) students chewed khat within the last 3 months. 247 (39.6%) participants reported alcohol drinking in their lifetime while only 138 (22.1%) students drank alcohol within the last 3 months. Regarding cigarette smoking, 26 (4.2%) of the total participants smoke within their lifetime and 20 (3.2%) smoke cigarettes within the last 3 months (see Figure 2).

### 3.4. Parental Neglect-Related Variables

Parental neglect among adolescents was assessed using the Adverse Childhood Experience Questionnaire in which the neglected part assessed physical and emotional neglect. Among the 624 adolescents who participated in this study, 334 (53.5%) participants answered yes to one or more questions among the total 10 items of the Adverse Childhood Experience Questionnaire and females account 190 (56.9%) of the total response. Among this figure of students experiencing parental neglect, more than half (175 (52.4%)) of them were grade 9 students (see Table 2).

### 3.5. Prevalence of Depression

**Figure 3:**
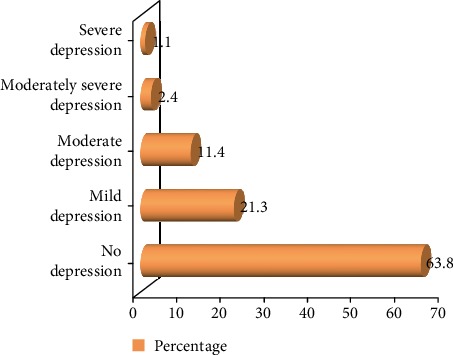
Characterization of depression severity among adolescents in governmental high schools at Aksum town, 2019 (*n* = 624).

The study showed that the prevalence of depression was 226 (36.2%) with 95% CI (32.3, 40.2). The prevalence rate was higher among grade 10 students since 110/296 (37.2%) of them met the screening criteria for depression which is higher when compared to 116/328 (35.4%) of them who met the screening criteria for depression in the study. According to the PHQ-9 severity classification from the total students under depression, 133 (21.3%) students lie in the mild depression category whereas 74 (11.4%), 15 (2.4%), and 7 (1.1%) students were found to have moderate, moderately severe, and severe depression, respectively (see Figure 3).

### 3.6. Association between Depression and Parental Neglect

Adverse Childhood Experience Questionnaire which assesses physical neglect, educational neglect, emotional neglect, and medical neglect was used to assess the main independent variable. Physical neglect refers to parents' negligence to provide adequately nutritious meals consistently, or it might mean that parents have abandoned their child. Educational neglect is a failure to provide a child with adequate education in the form of enrolling them in school or providing adequate homeschooling. Emotional neglect is consistently ignoring, rejecting, verbally abusing, teasing, withholding love, isolating, or terrorizing a child. Medical neglect is, in turn, the failure to provide appropriate health care for a child (although financially able to do so) [20].

The adverse childhood experience questionnaire was checked for colinearity between each item using the Pearson correlation coefficient at the *p* value of <0.05. As a result, there was no colinearity found between each item of the screening tool. A reliability test was conducted among the 10 items, and it has been found to have high reliability (Cronbach′s alpha = 0.83). After it is checked for colinearity, it was entered into logistic regression analysis and it is found to have a *p* value of <0.25 on bivariate analysis crude odds ratio (COR = 2.75, 95% CI (1.95, 3.89); *p* value = 0.000).

In addition to parental neglect, bivariate analysis was done for other explanatory variables for depression and the result revealed that explanatory variables, sex, family size, father's education, mother's education, social support, and current use of alcohol, were found to have a *p* value < 0.2. These factors were entered into multivariate logistic regression for further analysis to control confounding effects. As a result, being female, poor social support, mother's educational status, and parental neglect are found to be statistically significant for depression at a *p* value < 0.05. The odds of developing depression among those who had parental neglect were 2.61 times higher as compared to those who have no parental neglect (AOR = 2.61, 95% CI: 1.83, 3.72) (see Table 3).

### 3.7. Discussion on the Prevalence of Depression and Parental Neglect

The study revealed that the prevalence of depression was 36.2%. This result was in line with studies conducted in Addis Ababa (39.3%) among adolescents in governmental high schools [16] and Northern Iran 34% among high school and preuniversity adolescents using Beck's questionnaire [21].

However, the current finding for the study on depression was higher than that for the studies conducted among adolescents at Korea (20.6%) [22], Saudi Arabia (23.8%) [23], Egypt (28.6%) [24], Malaysia (10.3%) [25], and Trinidad (25.3%) [26]. The reason for the above difference might be due to difference in adolescent age which was only 13-19 in Trinidad [26], study populations who were only boys in Korea [22], the type of study conducted which was a large survey in Ethiopia [27], the screening tool which was BDI II in a study conducted in Saudi Arabia [23] and the children's depression inventory in a study in Malaysia [25], and the sample size which was 1373 in Egypt [24].

On the other hand, the finding of this study on the prevalence of depression was lower than that of a study conducted in Can Tho City, Vietnam (41.1%) [28], and Hong Kong, China (50%) [29]. This difference might be attributed to the time point of the study conducted which was a long-term study in Hong Kong [29], difference in study subjects in which only those adolescents who are abused physically and emotionally were studied, and difference in sample size in which a large sample size was used in Vietnam, i.e., 1159 students [28].

The above difference might also be due to differences in screening tools used to determine depression in which the Center for Epidemiology Studies Depression Scale (CES-D) was used in a study conducted in Can Tho City, Vietnam [28].

Regarding the severity of depression, the prevalence of mild depression was in line with a study conducted in Egypt which was 21.5%. However, the result of this study for moderate and severe depression is higher than that of a study conducted in Egypt which was 7.1% and 0%, respectively [24], and in Iran 5.7% and 0.3%, respectively [21].

The result of this study on mild depression is also found to be lower than that of a study conducted in Iran among high school and preuniversity adolescents which was 28% [21]. A possible reason for the difference might be difference in screening tools used to determine depression such as CES-D which was applied in Iran [21] and sample size which was 1373 in Egypt [24].

Furthermore, the result of this study revealed the prevalence of parental neglect among adolescents to be 53.5%. This result has been found consistent with the study conducted in Addis Ababa preparatory school which was 53.1% [16]. On the other hand, the result of this study regarding the prevalence of parental neglect is lower than that of a general population survey study done at Quezon City in Metro Manila which was 75% [30], 83.8% in a study conducted in Iran [31], and 70.7% in South Africa [32]. This might be due to the difference in the study populations who were adults and sample size which was 1068 in a study done at Quezon City in Metro Manila and 700 children in a study in Iran. This difference can also be due to the reason that they use different screening tools to assess parental neglect. However, the result of this study was higher than that of a study conducted at 26%. The possible reason for such difference might be sample size difference which was 8667 in Atlanta and study participants who were children in a study conducted in Atlanta [33].

### 3.8. Discussion on the Association between Depression and Parental Neglect

Parental neglect which is the main independent variable is found to be statistically associated with depression at a *p* value < 0.05. Analysis of the students' parental neglect with other explanatory variables was tried to control for confounding variables. After multivariate analysis, the strength of association between depression and parental neglect does not show a significant difference, i.e., COR = 2.75 and AOR = 2.61.

Students who were experiencing parental neglect were 2.61 times more likely to develop depression than those who did not experience parental neglect. This study is in line with a study conducted in Addis Ababa (AOR = 2.9) [16]. This may be because among the most common outcomes of neglect is the failure to succeed. Breakdown to succeed is a term that is normally applied to explain kids with a strange prototype of weight gain or weight loss or experiencing inadequate growth patterns (both mental and physical health) per a kid's age and developmental phase. This situation can occur when a child does not get sufficient diet or necessary medical consideration essential for appropriate bodily development [34], which may later hinder adolescents' overall physical health including mental health and lead them to depression.

In more tremendous cases, breakdown to succeed can also influence children over their entire existence course by really destructing their cognitive progress and their immune system due to inadequate calorie intake or lack of therapeutic consideration, making the child lose developmental milestones to a great extent and a great extent prone to poor health even after adulthood and give way to depression [34].

It might also be because a preponderance of neglected kids displays attachment disorder manifestations and finally forms timid connections even to their close families. This disturbed attachment to their primary caregiver alters their upcoming interaction with peers by making them emotional and physically isolated from others, and this in turn reduces the possibility of forming true relations. Moreover, as a result of their precedent abuse, neglected children experience that forming close relationships with others loses their control in life and exposes them by raising their susceptibility [35].

Neglectful parents and caregivers give poor interaction and positivity for their belongings which is linked to increased levels of shame called shame-proneness [36]. Shame-proneness may increase neglected adolescent's risk for depressive symptoms since they try to suppress such an aversive feeling. Shame suppression, in turn, may lead to sadness, social isolation and withdrawal, and lastly depression [37, 38].

The development of the brain may continue beyond adolescence age group. Therefore, neglectful experiences may impose a lasting effect on the continuing need for optimal conditions for development of some structures concerned with attention and emotional regulation, which contributes to the heightened occurrence of depression in victims [39].

The increased occurrence of depression in those who are experiencing parental neglect might also be due to the reason that neglected children show trouble in regulating their feeling and appreciating others' emotional expression and trouble in differentiating emotions which amplify their susceptibility for developing depression. Youths with a history of neglect during their early ages may also have stressful reminders which contributes to their current depressive state by suppressing and leads to deregulation of their emotion [40].

The higher prevalence of depression may also be a result of the injured hippocampus, as there are elevated levels of stress hormones such as cortisol due to increased stress levels in youths who had experienced neglect. This increased release of stress hormone is assumed to have an injury on the hippocampus, cortical area implicated in diverse brain function, and this, in turn, gives rise to developing depression in youths [41].

## 4. Conclusions and Recommendations

In the current study, the prevalence of depression is found to be high when compared to other populations. A significant and strong association is also determined between parental neglect and depression. During the data collection period, patients with a severe depression category were linked to a hospital and 24 of them got psychiatric evaluation for diagnosis and treatment accordingly. The result of this study has been submitted to stallholders for special consideration to depression and parental maltreatment towards adolescents. It is good if school teachers give emphasis to those students who seem psychologically unwell. It is better if school teachers exercise recommending such students to school psychologists. It is good to conduct a prospective cohort study to investigate the temporal relationship between factors and depression. It is good if Aksum University comprehensive hospital starts a campaign which will teach about the effect of parental neglect on the adolescent's mental health. Then, it is good to start clinic service for students who are psychologically unwell including consultation service.

## Figures and Tables

**Table 1:** Distribution of sociodemographic factors in high school students at Aksum town (*n* = 624).

Variable	Frequency	Percent (%)
Sex		
Male	285	45.7
Female	339	54.3
Age		
15	246	39.4
16	217	34.8
17	78	12.5
18	63	10.1
19	20	3.2
Grade		
Grade 9	328	52.6
Grade 10	296	47.4
Religion		
Orthodox	494	79.2
Muslim	101	16.2
Protestant	24	3.8
Other	5	.8
Family size		
1-5	372	59.6
>5	252	40.4
Residence		
Urban	422	67.6
Rural	202	32.4
Father's occupation		
Farmer	220	35.3
Labor work	32	5.1
Merchant	112	17.9
Government employee	158	25.3
Private employee	102	16.3
Mother's occupation		
Farmer	175	28.0
Labor work	38	6.1
Merchant	76	12.2
Government employee	107	17.1
Private employee	93	14.9
Housewife	135	21.6
Father's educational status		
Illiterate	83	13.3
1-4th grade	162	26.0
5-8th grade	143	22.9
9-12thgrade	112	17.9
Certificate & above	124	19.9
Mother's educational status		
Illiterate	176	28.2
1^st^-4^th^ grade	136	21.8
5^th^-8^th^ grade	124	19.9
9^th^-12^th^ grade	117	18.8
Certificate & above	71	11.4

**Table 2:** Distribution of Adverse Childhood Experience Questionnaire by sex and educational level among adolescents in the sampled governmental high schools in Aksum town, Ethiopia, 2019 (*n* = 624).

Variables	Male (*n*, %)	Female (*n*, %)	Grade 9 (*n*, %)	Grade 10 (*n*, %)	Total (*n*, %)
Physically hurt					
Yes	74 (11.9)	116 (18.6)	98 (15.7)	92 (14.7)	190 (30.4)
No	211 (33.8)	223 (35.7)	230 (36.9)	204 (32.7)	434 (69.6)
Hit you marks of injury					
Yes	34 (5.5)	58 (9.3)	48 (7.7)	44 (7)	92 (14.7)
No	251 (40.2)	281 (45)	280 (44.9)	252 (40.4)	532 (85.3)
Sexual abuse					
Yes	26 (4.2)	45 (7.2)	41 (6.6)	30 (4.8)	71 (11.4)
No	259 (41.5)	294 (47.1)	287 (46)	266 (42.6)	553 (88.6)
No love					
Yes	41 (6.6)	67 (10.7)	51 (8.2)	57 (9.1)	108 (17.3)
No	244 (39.1)	272 (43.6)	277 (44.4)	239 (38.3)	516 (82.7)
Not enough food or protection					
Yes	35 (5.6)	44 (7)	43 (6.9)	36 (5.8)	79 (12.7)
No	250 (40.1)	295 (47.3)	285 (45.7)	260 (41.7)	545 (87.4)
Divorced parents					
Yes	31 (5)	49 (7.8)	41 (6.6)	39 (6.2)	80 (12.8)
No	254 (40.7)	290 (46.5)	287 (46)	257 (41.2)	544 (87.2)
Abuse with gun or knife					
Yes	20 (3.2)	29 (4.6)	28 (4.5)	21 (3.4)	49 (7.9)
No	265 (42.5)	310 (49.7)	300 (48.1)	275 (44)	575 (92.1)
Live with alcoholic or drug user					
Yes	29 (4.6)	42 (6.7)	40 (6.4)	31 (5)	71 (11.4)
No	256 (41.1)	297 (47.6)	288 (46.1)	265 (42.5)	553 (88.6)
Depressed or attempted suicide household member					
Yes	19 (3)	28 (4.5)	31 (5)	16 (2.6)	47 (7.6)
No	266 (42.6)	311 (49.9)	297 (47.6)	280 (44.8)	577 (92.4)
Household member in prison					
Yes	41 (6.6)	46 (7.4)	50 (8)	37 (6)	87 (14)
No	244 (39)	293 (47)	278 (44.5)	259 (41.5)	537 (86)
Parental neglect					
Yes	144 (23.1)	190 (30.4)	175 (28)	159 (25.5)	334 (53.5)
No	141 (22.6)	149 (23.9)	153 (24.5)	137 (22)	290 (46.5)

**Table 3:** Bivariate and multivariate logistic analysis of factors associated with depression among adolescents in the sampled governmental high schools in Aksum town, Ethiopia, 2019 (*n* = 624).

Variable	Category	Depression		COR (95% CI)	AOR (95% CI)	*p* value
		Yes	No			
Sex	Male	83	202	1	1	
	Female	226	113	4.86 (1.82, 5.22)	1.48 (1.03, 2.13)	0.034

Family size	≤5	143	229	1	1	
	>5	83	169	0.79 (0.56, 1.10)	0.77 (0.53, 1.10)	0.150

Social support	Poor	107	149	1.86 (1.21, 2.88)	1.69 (1.07, 2.69)	0.026
	Moderate	77	140	1.43 (0.91, 2.24)	1.56 (0.97, 2.52)	0.067
	Good	42	109	1	1	

Current alcohol	Yes	98	149	1.28 (0.918, 1.784)	0.73 (.51, 1.06)	0.100
	No	128	249	1	1	

Mother's education	Illiterate	75	99	2.07 (1.13, 3.80)	2.21 (1.09, 4.49)	0.028
	1-4	49	86	1.56 (0.83, 2.93)	1.45 (0.70, 3.01)	0.317
	5-8	47	79	1.63 (0.861, 3.08)	1.67 (0.83, 3.35)	0.153
	9-12	36	82	1.20 (0.62, 2.31)	1.27 (.64, 2.56)	0.496
	College and above	19	52	1	1	

Father's education	Illiterate	35	46	1.81 (1.01, 3.24)	1.11(.56, 2.20)	0.768
	1-4	62	101	1.46 (0.89, 2.40)	0.92(.495, 1.706)	0.789
	5-8	56	87	1.53 (0.92, 2.55)	1.11 (0.61, 2.03)	0.730
	9-12	36	76	1.13 (0.65, 1.96)	1.00(.55, 1.83)	0.997
	College and above	37	88	1	1	

Parental neglect	Yes	156	178	2.75 (1.95, 3.89)	2.61 (1.83, 3.72)	0.000
	No	70	220	1	1	

## Data Availability

All the data included in the manuscript can be accessed from the corresponding author Mengesha Srahbzu upon request through email address of mengusew@gmail.com.
